# Size-Dependent Phagocytic Uptake and Immunogenicity of Gliadin Nanoparticles

**DOI:** 10.3390/polym12112576

**Published:** 2020-11-02

**Authors:** Mohammed S. Alqahtani, Rabbani Syed, Meshal Alshehri

**Affiliations:** 1 Department of Pharmaceutics, College of Pharmacy, King Saud University, Riyadh 11451, Saudi Arabia; 2 Nanomedicine & Biotechnology Research Unit, Department of Pharmaceutics, College of Pharmacy, King Saud University, Riyadh 11451, Saudi Arabia; rsyed@ksu.edu.sa; 3 Department of clinical laboratory sciences, College of Applied Medical Sciences, King Saud University, Riyadh 11451, Saudi Arabia; 437106442@student.ksu.edu.sa

**Keywords:** gliadin nanoparticles, immunogenicity, hemolysis, polymorphonuclear (PMN), phagocytosis

## Abstract

The main objective of the present study was to investigate the hemo and immune compatibility of gliadin nanoparticles as a function of particle size. Gliadin nanoparticles of different size were prepared using a modified antisolvent nanoprecipitation method. The hemolytic potential of gliadin nanoparticles was evaluated using in vitro hemolysis assay. Phagocytic uptake of gliadin nanoparticles was studied using rat polymorphonuclear (PMN) leukocytes and murine alveolar peritoneal macrophage (J774) cells. In vivo immunogenicity of gliadin nanoparticles was studied following subcutaneous administration in mice. Gliadin nanoparticles were non-hemolytic irrespective of particle size and hence compatible with blood components. In comparison to positive control zymosan, gliadin nanoparticles with a size greater than 406 ± 11 nm showed higher phagocytic uptake in PMN cells, while the uptake was minimal with smaller nanoparticles (127 ± 8 nm). Similar uptake of gliadin nanoparticles was observed in murine alveolar peritoneal macrophages. Anti-gliadin IgG antibody titers subsequent to primary and secondary immunization of gliadin nanoparticles in mice were in the increasing order of 406 ± 11 nm < 848 ± 20 nm < coarse suspension). On the other hand, gliadin nanoparticles of 127 ± 8 nm in size did not elicit immunogenic response. Phagocytosis and immunogenicity of gliadin nanoparticles are strongly influenced by particle size. The results of this study can provide useful information for rational design of protein-based nanomaterials in drug delivery applications.

## 1. Introduction

Polymeric micro and nano carriers prepared using synthetic and natural polymers are explored as carriers for drugs, vaccines, and diagnostic agents from organ to cellular level [1]. Biodegradable synthetic polymers such as poly lactic–*co*–glycolic acid (PLGA) have advantages of precise control of purity, composition, and safety [2]. However, some studies have reported that degradation products of PLGA polymer have the capacity to induce exaggerated inflammatory response [3,4]. Natural polymers derived from a plant source such as proteins have gained popularity compared to the synthetic variety due to biodegradability, biocompatibility, and economic and environmental friendliness [5]. Further, proteins have numerous surface functional groups for surface modification and can be prepared as films, fibers, hydrogels, and micro and nanoparticles for pharmaceutical applications. Gliadin, a hydrophobic protein extracted from wheat gluten, has been widely studied in food and pharmaceutical applications [6]. Its biocompatibility, hydrophobicity, and ability to modify and enhance drug release profile make it a potential drug carrier [7]. Furthermore, use of gliadin in medical scaffolds, nanofibers, films, and micro and nanoparticles showed adequate stability and flexibility [8,9,10,11]. Gliadin nanoparticles (GNPs) have been formulated for their applications in sustained drug release for various drugs including meletin, amoxicillin, polymethoxyflavons, resveratrol, paclitaxel, and carbazole [12,13,14,15]. Hence, during the development of gliadin-based particulate systems, understanding the interaction of nanoparticles with blood and immune cells is essential for safety [16,17,18]. One of the major limitations of parenterally administered particulates is the recognition by the immune cells of the reticuloendothelial system (RES), and subsequent clearance from the circulation without exerting the intended effect [19]. More importantly, parenterally injected particulates immediately interact with plasma and blood components. This interaction involves adsorption of major opsonin proteins such as immunoglobulins onto particle surfaces, which leads to phagocytic recognition and uptake [20]. According to the particle size and surface properties, particles may be taken up by immune cells in the blood stream by monocytes, platelets, leukocytes, and dendritic cells and in tissues by resident phagocytes including Kupffer cells in the liver, dendritic cells in lymph nodes, and macrophages and B-cells in the spleen. Various researchers have dedicated their efforts to the development of efficient strategies to overcome the immune system [21,22]. The influence of particle parameters such as size, shape, and surface properties on phagocytosis and immunogenicity was investigated using synthetic hydrophobic polymers [22,23]. In particular, the functional relationship between particle size and phagocytosis was more likely to have influenced cell surface interactions, resulting in immunological reaction. Micron sized particles were taken up by macrophages of liver and spleen through a receptor mediated process and engulfed by an actin-driven process [18,24]. These studies indicate that large size, hydrophobicity, and positive surface charge favors phagocytic uptake. However, little is known about the relationship between particle size and phagocytosis of natural hydrophobic proteins. For drug delivery applications intended to be administered systemically, the immunogenicity must be minimized to realize the potential of natural hydrophobic proteins [25,26]. The immunogenicity of hydrophobic plant proteins for instance legumin nanoparticles and zein microspheres was reported [27,28]. However, mechanistic details of influence of particle characteristics in relation to phagocytic uptake and immunogenicity are still to be determined. In the current study, we investigated the influence of particle size on blood compatibility, polymorphonuclear (PMN) cell uptake, macrophage phagocytic uptake, and in vivo immunogenicity of gliadin nanoparticles.

## 2. Materials and Methods

### 2.1. Materials

Gliadin from wheat, ethyl alcohol, 6-hydroxy coumarin, and trehalose dihydrate (anhydrous) was obtained from Sigma-Aldrich (St. Louis MO, USA). Sodium hydroxide, citric acid, sodium citrate, Pluronic F-127, a BCA assay kit (Pierce, Thermo Scientific, Chicago, IL, USA), Dulbecco’s modified Eagle’s media (DMEM, GIBCO^®^), fetal bovine serum (Atlanta Biologicals Inc., Lawrenceville, GA, USA), and penicillin/streptomycin (GIBCO™) were from Fisher Scientific Ltd. (Loughborough, UK). FITC tagged IgG was from Molecular Probes (Eugene, OR, USA). Mouse IgG was purchased from Santa Cruz Biotechnology (Dallas, TX, USA). Green fluorescent non-functionalized polystyrene particles were from Polyscience Inc. (Washington, PA, USA). Ficoll–Paque™ was purchased from GE health care (Piscataway, NJ, USA). All the other chemicals and solvents were analytical grade from Sigma-Aldrich Co. (St. Louis, MO, USA).

### 2.2. Cell Lines

Murine peritoneal macrophages (J774) were purchased from the American Type Culture Collection (ATCC, Manassas, VA). Media were supplemented with 10% heat inactivated fetal bovine serum and 1% penicillin/streptomycin. J774 cells were grown under standard cell culture conditions of 5% CO_2_ humidified air and 37 °C. Experiments were performed between passage numbers of 3 to 10. The 6-coumarin (fluorescent probe) encapsulated gliadin particles were used for uptake studies in J774 macrophage cells using flow cytometry (FACS, BD biosciences, San Jose, CA, USA).

### 2.3. Preparation of Gliadin Nanoparticles

Gliadin nanoparticles were prepared using a modified antisolvent nanoprecipitation method [29]. Low (0.025 g), medium (0.050 g), and high concentrations (0.1 g) of gliadin were used for preparation and optimization of nanoparticles with different sizes. Gliadin was dissolved in a mixture of ethanol and water (85% *v/v*). For dye-loaded nanoparticles, 6-coumarin (10 µg/mL) was added in the hydro alcoholic phase. Gliadin solution was added dropwise under constant application of ultrasonic energy (750 watts and 20 kHz frequency, Sonics^®^ USA) into 15 mL of citrate buffer (pH 7.4) containing (0.5% *w*/*v*) Pluronic F-127 for a period of 10 min. Subsequently, the nanoparticle suspension was kept on a magnetic stirrer at 300 rpm at room temperature until the ethanol was completely evaporated. After complete evaporation of ethanol, nanoparticles were purified to remove excess 6-coumarin and surfactant. The aqueous suspension of gliadin nanoparticles was purified by two cycles of differential centrifugation (3980× *g*, 50 min) using Amicon^®^ centrifugal filters (M.W. cut off 10k Da). After centrifugation, the supernatant was discarded, and the pellet was redispersed in 5 mL of buffer. Finally, 2% *w*/*v* trehalose was added to the aqueous nanoparticle suspension and lyophilized (Virtis^™^, Benchtop model, USA).

### 2.4. Characterization of Gliadin Nanoparticles

#### 2.4.1. Particle Size Analysis and Zeta Potential

Briefly, 10 mg of nanoparticles were dispersed in 2 mL of buffer then probe-sonicated and centrifuged at 10,000 rpm for 1 min. A 100 µL aliquot of supernatant was used for the determination of size and zeta potential using a Malvern Zetasizer-S 3600 (Malvern Instruments Inc., Southborough, MA, USA). Each sample was measured in triplicate, and the results are expressed as mean ± SD.

#### 2.4.2. Scanning Electron Microscopy (SEM) Analysis

SEM micrographs were obtained with a high-resolution scanning electron microscope (JSM-7500F, JEOL, Tokyo, Japan). A nanoparticle suspension (10 μL) was deposited directly onto a carbon grid, dried, and gold-coated under vacuum. Secondary electrons were collected after backscattering of the gold-coated samples attained by electron beams with a 10 kV acceleration voltage.

#### 2.4.3. Encapsulation Efficiency

About 5 mg of the nanoparticles were dispersed in 1 mL of purified water and centrifuged at 10,000 rpm for 10 min at 4 °C. An aliquot of the supernatant was diluted with ethanol and used for the determination of free coumarin by HPLC analysis (HPLC Beckman Coulter, Brea, CA, USA). The concentration of 6-coumarin was determined using gradient HPLC analysis (1 mM heptanesulfonic acid and acetonitrile 5% *v*/*v* for 3 min, 80% *v*/*v* for 11 min, and 5% *v*/*v* for 22 min) at a flow rate of 1 mL/min using a fluorescence detector. Fluorescence measurements were conducted at 450 and 490 nm for excitation and emission wavelengths, respectively. Furthermore, the nanoparticle matrix was digested with 85% *v/v* ethanol, and an aliquot was used for determination of gliadin concentration using standard protein assay, with gliadin as standard. From the digested nanoparticle matrix, an aliquot was diluted with ethanol, and encapsulated coumarin content was determined using the standard curve generated in 85% *v*/*v* ethanol. Free coumarin in the supernatant was subtracted from the amount in the nanoparticle. Encapsulation efficiency was calculated as % mg of drug loaded per mg of the protein (BCA assay) relative to the theoretical loading. Encapsulation efficiency was expressed as % mean of three experiments (± SD).

### 2.5. Hemolysis Assay

The hemolysis assay was performed using the method reported in the literature [30]. Briefly, fresh rabbit blood was collected in heparinized tubes from the Animal Care and Use Centre, College of Pharmacy, King Saud University (Riyadh, Saudi Arabia). Subsequently, blood samples were stabilized in 0.3 mL acid citrate dextrose (ACD) to prevent clotting and stored at 4 °C until further use. Triton X-100 with a final concentration of 1% and saline (0.9% *w*/*v* NaCl) were used as positive and negative controls. Different sized gliadin nanoparticles in distilled water, with the final mass concentrations of particles in the tubes ranging from 50 to 2000 mg/mL, were incubated with ACD stabilized blood for 30 min at 37 °C in an orbital shaker (100 rpm). Following the incubation, the tubes were centrifuged for 15 min at room temperature. The supernatants were mixed at a 1:1 ratio with CMH reagent and analyzed, and the optical density of supernatant was measured at 545 nm. The total hemoglobin concentration of heparinized whole blood was measured based on a hemoglobin concentration standard curve.

### 2.6. Polymorphonuclear Cell Uptake

The polymorphonuclear (PMN) cell uptake assay procedure was performed according to the method reported in the literature [31]. Briefly, PMN cells were isolated from freshly collected rabbit blood from the Animal Care and Use Centre, College of Pharmacy, King Saud University (Riyadh, Saudi Arabia), using the Ficoll–Hypaque density gradient technique. Different sized blank gliadin nanoparticles were diluted at a 1:10 (*w*/*v*) ratio in PBS pH 7.4 before addition to the microtiter plate (final concentration, 250 μg/100 μL). To measure the chemiluminescence (CL), 50 μL of PMN cell suspension (5 × 10^6^ cells/mL) was added to each microtiter plate. The nanoparticles and positive control zymosan particles were added to the wells, and chemiluminescence was determined for 180 min. In each experiment, 1 μm nanoparticle and zymosan particle uptake was used as control and internal standard (100% uptake). The AUC of the different sized particles was expressed in % of the AUC of control particles.

### 2.7. FITC Tagged IgG Adsorption

Blank polystyrene (1 μm, 1% *w*/*v*), and gliadin nanoparticles (848, 406, and 127 nm, 1% *w*/*v*) were incubated with 2 mL of FITC-labeled IgG solution (0.01 M phosphate buffered saline pH 7.4) at 37 °C on an rotating shaker (100 rpm) for 2 h. The suspension was then centrifuged at 12,000 rpm for 10 min. The supernatant was removed, and the nanoparticles were resuspended in 2 mL of phosphate buffer at pH 7.4. The centrifugation followed by the washing step was repeated three times. The adsorbed IgG was determined by measuring the fluorescence values of FITC using a spectrofluorometer (JASCO FP-8300, JASCO, Japan) at excitation and emission wavelengths of 495 and 520 nm, respectively. Furthermore, supernatant was used at each washing step for determination of free IgG. Adsorbed IgG was estimated indirectly by subtracting the free IgG present in the supernatant after washings.

### 2.8. In Vitro Phagocytic Uptake Assay

A phagocytic assay was used for the J774 cell line (murine peritoneal macrophages). For IgG-opsonized and non-opsonized uptake experiments, 6-coumarin-loaded gliadin nanoparticles were used. Green fluorescent non-functionalized polystyrene particles (1 μm, 1% *w*/*v*) were used as the positive control. For the opsonization study, polystyrene particles or gliadin nanoparticles in buffer were incubated with IgG (1mg/mL) for 30 min prior to incubation with J774 cells. In the case of the non-opsonized uptake study, particles were used without pre incubation with IgG. Cells were seeded in a 12-well plate at a concentration of 5 × 10^4^ cells/well and were allowed to adhere overnight. Polystyrene particles and gliadin nanoparticles at a concentration of 2 mg/mL were used for phagocytic uptake experiments. Particles were incubated with J774 cells for 30 min at 37 °C. At the end of incubation, cells were scrapped from the wells and washed three times with cold buffer. Samples were analyzed using flow cytometry (FACS, BD biosciences, San Jose, CA, USA), and mean fluorescence intensity of the cells was recorded at 10,000 events.

### 2.9. In Vivo Immunization of Mice with Gliadin Nanoparticles

All procedures performed in animals were approved by the Animal Care and use committee of King Saud University (Ethics Ref. No. KSU-SE-20-10) and performed according to the National Institutes of Health Guidelines for the Care and Use of Laboratory Animals. Female BALB/c mice were used for in vivo immunogenicity investigation. Mice were divided into five groups of four animals each. Gliadin coarse suspension was used as positive control. The saline group was used as a negative control. Nanoparticle formulations were administered subcutaneously in physiological saline (0.9% *w*/*v* sodium chloride) at a dose of 100 µg/50 µL (equivalent gliadin concentration). Blood samples were collected on days 14 and 28 after primary and secondary immunization from the retro-orbital plexus. Serum samples were analyzed for anti-gliadin IgG antibody titers using a standardized sandwich ELISA method. Briefly, a 96-well plate was coated overnight with gliadin (1% *w*/*v*) in 90% *v*/*v* ethanol at 4 °C. After overnight incubation, plates were washed using buffer (1% tween 20 in PBS) and blocked with 1% bovine serum albumin (BSA) for 1 h at 37 °C. In this step, all the procedures were performed at 37 °C. Plates were washed using buffer, followed by serum incubation (1/16 dilution) for 1 h. Serum was removed followed by washing with buffer and incubation of goat anti-mouse IgG conjugated to horseradish peroxidase for 1 h, and the washing step was repeated. TMB substrate was added followed by 5 min incubation, and the reaction was stopped by 1M H_2_SO_4_. The absorbance was recorded using a UV-visible spectrophotometer (JASCO, Japan) at 450 nm.

### 2.10. Data Analysis

All the experiments were performed in triplicate, and the results are expressed as mean ± SD. One-way ANOVA (GraphPad software, CA, USA) was used to compare the different groups, and the results were considered to be significant at *p* < 0.05.

## 3. Results

### 3.1. Preparation of Gliadin Nanoparticles

**Figure 1:**
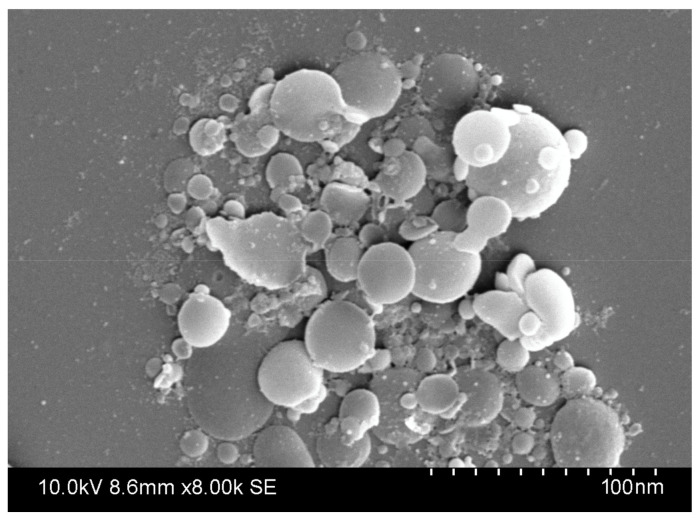
Scanning electron microscopy (SEM) images of gliadin nanoparticles.

Gliadin nanoparticles were prepared and optimized using a modified antisolvent nanoprecipitation method. The mean diameter of the blank gliadin nanoparticles ranged from 127 to 848 nm (Table 1). Irrespective of particle size, zeta potential of blank gliadin nanoparticles was −26 ± 2 mV. The coumarin encapsulated gliadin nanoparticles size ranged from 132 to 912 nm. Different sized coumarin-loaded gliadin nanoparticles have zeta potential of −19 ±5 mV (Table 2). Encapsulation efficiency of coumarin-loaded gliadin nanoparticles was 90 ± 3%. The green fluorescent compound 6-coumarin was used for characterization of uptake of gliadin nanoparticles in J774 (murine peritoneal macrophage) cells. The morphological characterization of nanoparticles using SEM microscopy (Figure 1) revealed a spherical shape with smooth surface morphology.

### 3.2. Hemolysis Assay

**Figure 2:**
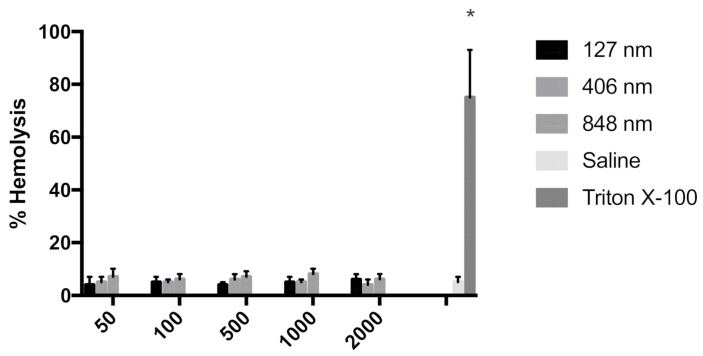
In vitro hemolysis test results of gliadin nanoparticles of varying size. Saline was used as a negative control, and 1% Triton X-100 was used as a positive control. Results are indicative of triplicate samples (n = 3, ±SEM). Hemolysis was unaffected by gliadin particle size (* p < 0.001).

From the results it was evident that irrespective of the particle size, gliadin nanoparticles were found be compatible with blood components (Figure 2). From the data it was imperative that concentration of surfactant and gliadin that was used for preparation of gliadin nanoparticles was hemocompatible.

### 3.3. Polymorphonuclear (PMN) Cell Uptake

**Figure 3:**
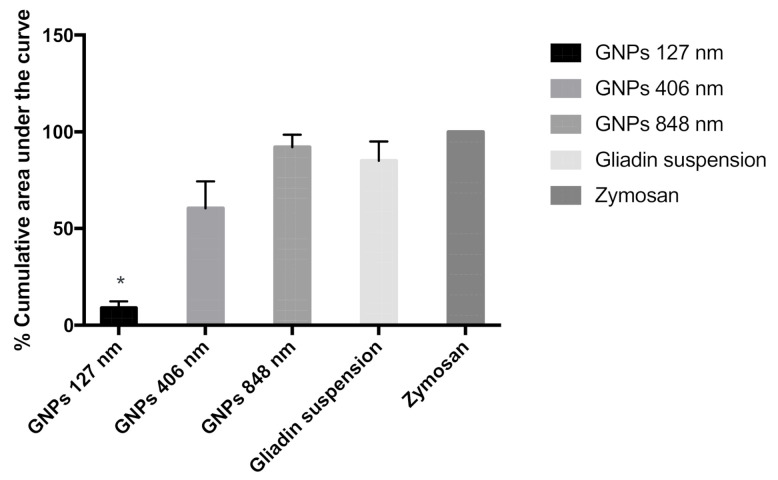
Influence of particle size on the uptake of polymorphonuclear (PMN) cells. The percentage area under the curve for chemiluminescence accumulated over the 90 min time interval was calculated with respect to positive control zymosan (100%), (n = 4, ±SEM). * indicates significance at (*p* < 0.05).

Polymorphonuclear (PMN) cells were used for uptake characterization of gliadin nanoparticles. Chemiluminescence-based studies indicate that the extent of uptake of gliadin nanoparticles is size-dependent (Figure 3). It was evident from the results that smaller the particle size, the lesser the uptake. In comparison to positive control zymosan, uptake was lowest with 127 nm and highest in the case of 848 and 406 nm particles. However, no significant difference was observed between 848 and 406 nm particles.

### 3.4. FITC Tagged IgG Adsorption

The adsorption profile of fluorescently tagged IgG on polystyrene and gliadin nanoparticles was investigated. As expected, a significant amount of IgG was adsorbed onto polystyrene nanoparticles through hydrophobic interactions. On the other hand, the least IgG adsorption was observed with 127 nm sized gliadin nanoparticles. Among gliadin nanoparticles, although statistically not significant, more IgG was adsorbed onto 406 nm particles in comparison to 848 nm particles.

### 3.5. In Vitro Phagocytic Uptake Assay

**Figure 4:**
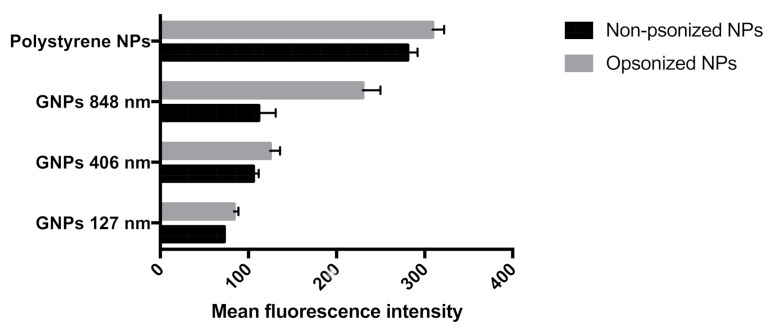
In vitro uptake of different gliadin nanoparticle sizes and polystyrene (1 μm) by J774 cells (mouse peritoneal macrophages). Phagocytic uptake is represented as mean fluorescence intensity. Each value represents mean ±SD of four independent experiments.

The influence of particle size on phagocytic uptake of murine peritoneal macrophage cells (J774 cells) is presented in Figure 4. In the case of polystyrene particles, significant difference in uptake between opsonized and non-opsonized particles was observed. A similar uptake pattern was observed within the tested particle sizes of gliadin nanoparticles, except in 127 nm particles. However, in the case of opsonized particles, significant difference in uptake was observed between polystyrene particles and 406 and 127 nm gliadin nanoparticles. In contrast, significant difference in uptake was observed between non-opsonized polystyrene particles and gliadin nanoparticles. Significant difference in uptake was observed for the non-opsonized gliadin nanoparticles between 848 and 127 nm.

### 3.6. In Vivo Immunogenicity of Gliadin Nanoparticles

**Figure 5:**
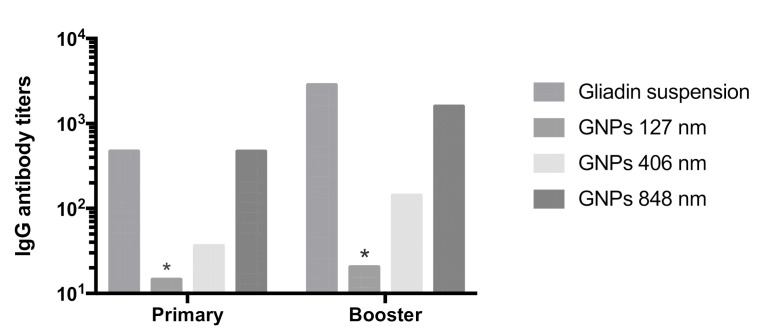
Anti-gliadin IgG antibody responses in BALB/c mice after subcutaneous injections of different gliadin particles. Titer value represents the lowest dilution of serum at which optical density is similar to saline (n = 4, ±SEM). * Non-immunogenic, similar to saline *p* < 0.05.

Production of anti-gliadin IgG antibodies and titers following subcutaneous administration of gliadin nanoparticles on days 14 and 28 after primary and booster doses is presented in Figure 5. Immunogenicity studies in mice showed that only smaller gliadin nanoparticles (127 nm) did not produce any IgG anti-gliadin antibodies and were therefore comparable to negative control. On the other hand, larger particles as well as coarse suspension produced a significant immune response compared to saline control.

## 4. Discussion

Although nanoparticles using water-soluble proteins have been widely reported in the literature, very little is known about the safety of hydrophobic protein nanoparticles especially for parenteral drug delivery applications [32]. The optimal size for a nanoparticulate formulation is an important criterion to overcome immunogenicity of hydrophobic protein nanoparticles. Moreover, controlling batch-to-batch uniformity, purity, and reproducibility of the composition is also challenging. We prepared different sized particles near to the defined size by altering the gliadin concentration using a modified antisolvent nanoprecipitation method developed in our laboratory. Our long-term goal is to develop gliadin-based nanoparticles for parenteral drug delivery applications. The parenteral route of administration is the most effective route for drug delivery especially for the active pharmaceutical agents with poor bioavailability or narrow therapeutic index [33]. After systemic administration, nanoparticles interact with red blood cells. Interaction of nanoparticles with red blood cells may induce damage, leading to leakage of the iron containing protein hemoglobin into the blood stream, which could potentially lead to life-threatening conditions such as anemia [17]. This phenomenon is referred to hemolysis. Several studies have demonstrated the hemolytic activities of cationic charged particles, while negative and anionic surfaces were shown to be non-hemolytic [34,35]. In the present study, gliadin nanoparticles were non-hemolytic irrespective of particle size. Pluronic F-127, as a surfactant, was used at a low concentration of 0.5% w/v in the preparation of gliadin nanoparticles, as this concentration is known to be safe and non-hemolytic [36]. The physicochemical properties of the nanoparticles such as composition, size, charge, hydrophobicity, and surface functional groups influence the extent of uptake, opsonization, and clearance by macrophages of the reticuloendothelial system (RES). Opsonization is a process by which a foreign organism or particle becomes covered with opsonin proteins [20]. Among plasma proteins, albumin and fibrinogen dominate the particle surface but are higher affinity proteins are subsequently displayed including immunoglobulins, laminin, fibronectin, C-reactive protein, and type-I collagen. Adsorption of opsonin proteins determines the subsequent particle uptake by various cells of the blood components and immune system. After opsonization, the proteins adsorbed on the nanoparticle surface interact with the monocytes and various subsets of macrophages and induce their cell uptake by phagocytosis. Depending on the nature of the complement protein adsorbed on the nanoparticle surface, one of the several pathways can be activated, including the classical pathway of antigen-antibody binding, the antibody independent pathway, or the lectin pathway [23]. Regardless of the pathway, the particles at the end are taken up by phagocytes, and the degraded particles are presented to the immune system. Depending on the antigenicity of the protein fragments, they may induce a humoral or cell immune response [17]. Previous studies have demonstrated that larger particles are taken up more efficiently than smaller particles of the same composition and surface properties [37]. Nanoparticles with a size ranging from 50 to 200 nm are generally preferred to prolong systemic circulation time, whereas nanoparticles of >500 nm are mainly removed via the reticuloendothelial system [23]. In the present work, uptake studies were performed using polymorphonuclear (PMN) leukocyte cells, as anticipated larger size gliadin nanoparticles were taken up more efficiently than smaller particles. Similar size-dependent uptake of polystyrene microspheres has been reported by Simon et al. using human blood neutrophils [38]. Furthermore, it has been shown that uptake of polystyrene microspheres increased with increase in particle size. Adsorption of plasma proteins such as immunoglobulin (IgG) onto nanoparticles occurs instantly as the particles enter the blood stream [39]. Subsequently, the effective size and charge of nanoparticles leads to macrophage uptake. In our study, adsorption of IgG was higher in the case of larger size polystyrene particles and gliadin nanoparticles as opposed to smaller particles. Similar observations were previously reported, which showed a lower protein adsorption with smaller nanoparticles (50 nm) compared to that of larger nanoparticles (≥200 nm) [21,39]. Generally, immune cells recognize nanoparticles based on their surface properties. The preparation process and purification treatment were suggested to cause thermal/chemical stress, which leads to conformational changes in the protein structure. Such confirmational changes can affect the immunogenic properties of native gliadin. Moreover, size uniformity and distribution determine the potential reactivity of gliadin nanoparticles towards IgG. Large particles with large polydispersity index (PDI) undergo rapid aggregation that transforms them into more stable thermodynamic states once they are brought into contact with a physiological medium. On the other hand, it is common to observe that smaller monodisperse particles become stable in the biological media due to the electrosteric stabilizing effect of adsorbed surfactant. Hence, the distributions of hydrophilic surfactant on the surface of nanoparticles may also be an important parameter affecting their immunogenicity through masking the surface epitopes. The presence of Pluronic on gliadin nanoparticle surfaces provides steric repulsion and hence prevents adsorption of IgG onto smaller sized gliadin nanoparticles. Although an equivalent concentration of surfactant was employed for preparation of different sized gliadin nanoparticles, larger sized particles tend to have highly hydrophobic surfaces for hydrophobic interactions. This could have resulted in the adsorption of IgG onto larger sized nanoparticles. In the case of smaller sized gliadin nanoparticles, a free hydrophobic surface is less available as less gliadin is used in comparison to larger sized particles. In the case of polystyrene microspheres, since they were non-functionalized, such highly hydrophobicity favors adsorption of IgG through hydrophobic interactions [18]. Subsequently, adsorption of IgG onto the surface of nanoparticles induces cell uptake by phagocytosis. Phagocytosis is also influenced by other factors including surface hydrophobicity and charge in addition to size [17]. The present study showed a higher uptake of large sized polystyrene and gliadin nanoparticles by J774 cells in comparison to small sized particles. Since polystyrene particles were more hydrophobic, higher uptake was observed in both opsonized and non-opsonized particles. Larger sized particles have relatively highly hydrophobic surface area and hence were taken up more efficiently by macrophages. However, uptake of gliadin nanoparticles was less in comparison to polystyrene particles. It has been reported that particles with sizes less than 0.5 µm are mainly taken up by macro-pinocytosis, while large sized particles are taken up by phagocytosis and are more likely to induce an immune response [40]. Another explanation was that the lower uptake of small sized particles could be due to extensive exocytosis. Non-opsonized particles are capable of adsorbing albumin from fetal bovine serum in the cell culture media. Our results can be further explained with the findings of Champion et al. [18], who postulated that intermediate sized particles are able to make better contact with the cell membrane than smaller or larger sized particles. Additionally, IgG adsorption leads to receptor mediated macrophage uptake through Fc receptors. Attachment and internalization of IgG-opsonized microspheres increased in comparison to non-opsonized counterparts. Further, intermediate sized microspheres have better contact with the cell membrane in comparison to smaller and larger sized particles. One explanation for these differences in the uptake could be the fact that differently sized particles have a remarkably different distribution, permeation, and stability in the interstitial fluid. Ultimately, the uptake of nanoparticles by immune cells such as neutrophils, monocytes, macrophages, and dendritic cells mediates an innate immune response. On the other hand, humoral immune responses are mediated by antibodies produced by B-cells or specifically activated T-cells. In this context, we investigated the immunogenicity of gliadin nanoparticles as a function of size using in vivo experiments. The in vivo results showed that production of anti-gliadin antibodies in mice was dependent on gliadin particle size. These differences on the antibody response elicited by the different sizes is mainly due to quantitative differences in the ability of macrophages to phagocytose a particle mass, depending on the size of the formulation administered, and to differences in the ability of the different particles to reach the lymphoid tissues, where a specific type of immune responses is induced [41]. Protein microspheres with a dimension greater than 1 µm allow them to act as a depot system resulting in continuous antigen release and prolonged antigen presentation. In one study, the immunogenicity of zein microspheres following intradermal administration in mice was investigated [28]. The authors concluded that zein microspheres were immunogenic and hence not suitable for drug and vaccine delivery applications due to the large size of blank microspheres (1356 ±36 nm), which are responsible for macrophage uptake, degradation, and presentation of degradation products to lymphocytes for the production of anti-zein antibodies. In another study, a higher molecular weight polyvinylpyrrolidone (PVP) surfactant was utilized in the preparation of protein particles, a type 2, T-cell independent (TI) antigen [42]. Hence, larger particle size in combination with higher molecular weight PVP could have resulted in the production of zein antibodies. Based on similar studies, legumin nanoparticles were investigated for their immunogenicity [27]. The authors reported that native legumin was immunogenic, but legumin nanoparticles were not. It was postulated that chemical cross linking of legumin nanoparticles during preparation could have masked the antigenic epitopes of legumin. Subsequently, the clearance of legumin nanoparticles by macrophages and incomplete degradation led to insufficient presentation of antigen to lymphocytes. Our result was consistent with such findings, and we reasoned that insufficient antigen presentation to lymphocytes could have occurred with 127 nm sized gliadin nanoparticles. In the case of large sized gliadin particles and coarse suspension, adsorption of IgG onto particles perhaps led to much higher phagocytic uptake, production of antigliadin antibodies, and efficient antigen presentation to lymphocytes. Taken together, the in vitro and in vivo studies demonstrated that phagocytic uptake and immunogenicity of gliadin nanoparticles is strongly dependent on particle size. Therefore, the findings of this study demonstrated the optimal particle size for the development of non-immunogenic gliadin nanoparticles for drug delivery applications.

## 5. Conclusions

Gliadin nanoparticles were compatible with blood irrespective of particle size. Immunoglobulin G adsorption, polymorphonuclear cells, and in vitro peritoneal macrophage uptake was strongly dependent on particle size. Immunogenicity studies showed that only smaller gliadin nanoparticles were non-immunogenic. On the other hand, gliadin coarse suspension as well as large particles were immunogenic. The results of this study demonstrated the potential application of gliadin nanoparticles for drug delivery applications.

## Figures and Tables

**Table 1:** Particle size distribution of gliadin nanoparticles of different treatment groups (n = 3, ±SD). * Polydispersity index.

Sample #	Particle Size (nm)	PI *
1.	848 ± 20	0.361 ± 0.05
2.	406 ± 11	0.243 ± 0.06
3.	127 ± 8	0.204 ± 0.02

**Table 2:** Particle size distribution of 6-coumarin encapsulated gliadin nanoparticles (n = 3, ±SD). * Polydispersity index.

Sample #	Particle Size (nm)	PI *
1.	912 ± 18	0.31 ± 0.08
2.	495 ± 12	0.259 ± 0.01
3.	132 ± 7	0.232 ± 0.03

## References

[1] Couvreur P., Vauthier C. (2006). Nanotechnology: Intelligent design to treat complex disease. Pharm. Res..

[2] Peer D., Karp J.M., Hong S., Farokhzad O.C., Margalit R., Langer R. (2007). Nanocarriers as an emerging platform for cancer therapy. Nat. Nanotechnol..

[3] Wang H.J., Lin Z.X., Liu X.M., Sheng S.Y., Wang J.Y. (2005). Heparin-loaded zein microsphere film and hemocompatibility. J. Control. Release.

[4] Podaralla S., Perumal O. (2012). Influence of formulation factors on the preparation of zein nanoparticles. AAPS PharmSciTech.

[5] Reddy N., Yang Y. (2011). Potential of plant proteins for medical applications. Trends Biotechnol..

[6] Mehanna M., Mneimneh A. (2020). Updated but not outdated “Gliadin”: A plant protein in advanced pharmaceutical nanotechnologies. Inter. J. Pharm..

[7] Arangoa M.A., Campanero M.A., Renedo M.J., Ponchel G., Irache J.M. (2001). Gliadin nanoparticles as carriers for the oral administration of lipophilic drugs. Relationships between bioadhesion and pharmacokinetics. Pharm. Res..

[8] Sripriyalakshmi S., Jose P., Ravindran A., Anjali C. (2014). Recent trends in drug delivery system using protein nanoparticles. Cell Biochem. Biophys..

[9] Urade R., Sato N., Sugiyama M. (2018). Gliadins from wheat grain: An overview, from primary structure to nanostructures of aggregates. Biophys. Rev..

[10] Xu Y., Li J.-J., Yu D.-G., Williams G.R., Yang J.-H., Wang X. (2017). Influence of the drug distribution in electrospun gliadin fibers on drug-release behavior. Eur. J. Pharm. Sci..

[11] Zhang Y., Yu W., Ba Z., Cui S., Wei J., Li H. (2018). 3D-printed scaffolds of mesoporous bioglass/gliadin/polycaprolactone ternary composite for enhancement of compressive strength, degradability, cell responses and new bone tissue ingrowth. Int. J. Nanomed..

[12] Yang Y.-Y., Zhang M., Liu Z.-P., Wang K., Yu D.-G. (2018). Meletin sustained-release gliadin nanoparticles prepared via solvent surface modification on blending electrospraying. Appl. Surf. Sci..

[13] Umamaheshwari R., Ramteke S., Jain N.K. (2004). Anti-Helicobacter pylori effect of mucoadhesive nanoparticles bearing amoxicillin in experimental gerbils model. AAPS Pharm..

[14] Davidov-Pardo G., Joye I.J., McClements D.J. (2015). Encapsulation of resveratrol in biopolymer particles produced using liquid antisolvent precipitation. Part 1: Preparation and characterization. Food Hydrocoll..

[15] Wang X. Assessing the Cytotoxicity of Newly Developed Gliadin Nanoparticles Loading Polymethoxyflavones. https://rucore.libraries.rutgers.edu/rutgers-lib/51492/.

[16] Dobrovolskaia M.A., Aggarwal P., Hall J.B., McNeil S.E. (2008). Preclinical studies to understand nanoparticle interaction with the immune system and its potential effects on nanoparticle biodistribution. Mol. Pharmaceutics.

[17] Dobrovolskaia M.A., McNeil S.E. (2007). Immunological properties of engineered nanomaterials. Nat. Nanotechnol..

[18] Champion J.A., Walker A., Mitragotri S. (2008). Role of particle size in phagocytosis of polymeric microspheres. Pharm. Res..

[19] Gaur U., Sahoo S.K., De Tapas K., Ghosh P.C., Maitra A., Ghosh P. (2000). Biodistribution of fluoresceinated dextran using novel nanoparticles evading reticuloendothelial system. Inter. J. Pharm..

[20] Owens III D.E., Peppas N.A. (2006). Opsonization, biodistribution, and pharmacokinetics of polymeric nanoparticles. Inter. J. Pharm..

[21] Lundqvist M., Stigler J., Elia G., Lynch I., Cedervall T., Dawson K.A. (2008). Nanoparticle size and surface properties determine the protein corona with possible implications for biological impacts. Proc. Natl. Acad. Sci. USA.

[22] Sharma G., Valenta D.T., Altman Y., Harvey S., Xie H., Mitragotri S., Smith J.W. (2010). Polymer particle shape independently influences binding and internalization by macrophages. J. Control. Release.

[23] Vonarbourg A., Passirani C., Saulnier P., Benoit J.-P. (2006). Parameters influencing the stealthiness of colloidal drug delivery systems. Biomaterials.

[24] Ziv O., Avtalion R.R., Margel S. (2008). Immunogenicity of bioactive magnetic nanoparticles: Natural and acquired antibodies. J. Biomed. Mater. Res. Part.

[25] Decuzzi P., Godin B., Tanaka T., Lee S.-Y., Chiappini C., Liu X., Ferrari M. (2010). Size and shape effects in the biodistribution of intravascularly injected particles. J. Control. Release.

[26] Elzoghby A.O., Samy W.M., Elgindy N.A. (2012). Protein-based nanocarriers as promising drug and gene delivery systems. J. Control. Release.

[27] Mirshahi T., Irache J., Nicolas C., Mirshahi M., Faure J., Gueguen J., Hecquet C., Orecchioni A. (2002). Adaptive immune responses of legumin nanoparticles. J. Drug Target..

[28] Hurtado-López P., Murdan S. (2006). An investigation into the adjuvanticity and immunogenicity of zein microspheres being researched as drug and vaccine carriers. J. Pharm. Pharmacol..

[29] Sharma K., Deevenapalli M., Singh D., Chourasia M.K., Bathula S.R. (2014). Preparation and characterization of paclitaxel-loaded gliadin nanoparticles. J. Biomater. Tissue Eng..

[30] Lai L., Guo H. (2011). Preparation of new 5-fluorouracil-loaded zein nanoparticles for liver targeting. Inter. J. Pharm..

[31] Potter T.M., Skoczen S.L., Rodriguez J.C., Neun B.W., Ilinskaya A.N., Cedrone E., Dobrovolskaia M.A., McNeil S.E. (2018). In Vitro Analysis of Nanoparticle Effects on the Zymosan Uptake by Phagocytic Cells. Characterization of Nanoparticles Intended for Drug Delivery.

[32] Lohcharoenkal W., Wang L., Chen Y.C., Rojanasakul Y. (2014). Protein nanoparticles as drug delivery carriers for cancer therapy. BioMed Res. Inter..

[33] Shetab Boushehri M.A., Dietrich D., Lamprecht A. (2020). Nanotechnology as a Platform for the Development of Injectable Parenteral Formulations: A Comprehensive Review of the Know-Hows and State of the Art. Pharmaceutics.

[34] Domański D., Klajnert B., Bryszewska M. (2004). Influence of PAMAM dendrimers on human red blood cells. Bioelectrochemistry.

[35] Malik N., Wiwattanapatapee R., Klopsch R., Lorenz K., Frey H., Weener J., Meijer E., Paulus W., Duncan R. (2000). Dendrimers: Relationship between structure and biocompatibility *in vitro*, and preliminary studies on the biodistribution of 125I-labelled polyamidoamine dendrimers *in vivo*. J. Control. Release.

[36] Lowe K.C., Furmidge B.A., Thomas S. (1995). Haemolytic properties of pluronic surfactants and effects of purification. Artif. Cells, Blood Substitutes, Biotechnol..

[37] Fang C., Shi B., Pei Y.-Y., Hong M.-H., Wu J., Chen H.-Z. (2006). In vivo tumor targeting of tumor necrosis factor-α-loaded stealth nanoparticles: Effect of MePEG molecular weight and particle size. Eur. J. Pharm. Sci..

[38] Simon S.I., Schmid-Schönbein G. (1988). Biophysical aspects of microsphere engulfment by human neutrophils. Biophy. J..

[39] Cedervall T., Lynch I., Lindman S., Berggård T., Thulin E., Nilsson H., Dawson K.A., Linse S. (2007). Understanding the nanoparticle–protein corona using methods to quantify exchange rates and affinities of proteins for nanoparticles. Proc. Natl. Acad. Sci. USA.

[40] Xiang S.D., Scholzen A., Minigo G., David C., Apostolopoulos V., Mottram P.L., Plebanski M. (2006). Pathogen recognition and development of particulate vaccines: Does size matter?. Methods.

[41] Kanchan V., Panda A.K. (2007). Interactions of antigen-loaded polylactide particles with macrophages and their correlation with the immune response. Biomaterials.

[42] Naim J.O., van Oss C.J. (1992). The effect of hydrophilicity-hydrophobicity and solubility on the immunogenicity of some natural and synthetic polymers. Immunol. Investig..

